# Inhibition of Marek’s Disease Virus Replication and Spread by 25-hydroxycholesterol and 27-hydroxycholesterol In Vitro

**DOI:** 10.3390/v15081652

**Published:** 2023-07-29

**Authors:** Nitin Kamble, Vishwanatha R. A. P. Reddy, Ben Jackson, Faisal R. Anjum, Chidiebere C. Ubachukwu, Ajit Patil, Shahriar Behboudi

**Affiliations:** Avian Immunology Group, The Pirbright Institute, Pirbright, Woking, Surrey GU24 0NE, UKvishi.reddy@pirbright.ac.uk (V.R.A.P.R.); faisal.anjum@pirbright.ac.uk (F.R.A.); chidiebere.ubachukwu@pirbright.ac.uk (C.C.U.); drajitpatil29@gmail.com (A.P.)

**Keywords:** Marek’s disease virus, virus replication, IFN-stimulated genes, 25-hydroxycholesterol, 27-hydroxycholesterol, CH25H, CYP27A1

## Abstract

Marek’s disease virus (MDV) causes a deadly lymphoproliferative disease in chickens, resulting in huge economic losses in the poultry industry. It has been suggested that MDV suppresses the induction of type I interferons and thus escapes immune control. Cholesterol 25-hydroxylase (CH25H), a gene that encodes an enzyme that catalyses cholesterol to 25-hydroxycholesterol (25-HC), is an interferon-stimulating gene (ISG) known to exert antiviral activities. Other oxysterols, such as 27-hydroxycholesterols (27-HC), have also been shown to exert antiviral activities, and 27-HC is synthesised by the catalysis of cholesterol via the cytochrome P450 enzyme oxidase sterol 27-hydroxylase A1 (CYP27A1). At 24 h post infection (hpi), MDV stimulated a type I interferon (IFN-α) response, which was significantly reduced at 48 and 72 hpi, as detected using the luciferase assay for chicken type I IFNs. Then, using RT-PCR, we demonstrated that chicken type I IFN (IFN-α) upregulates chicken CH25H and CYP27A1 genes in chicken embryo fibroblast (CEF) cells. In parallel, our results demonstrate a moderate and transient upregulation of CH25H at 48 hpi and CYP27A1 at 72hpi in MDV-infected CEF cells. A significant reduction in MDV titer and plaque sizes was observed in CEFs treated with 25-HC or 27-HC in vitro, as demonstrated using a standard plaque assay for MDV. Taken together, our results suggest that 25-HC and 27-HC may be useful antiviral agents to control MDV replication and spread.

## 1. Introduction

Marek’s disease virus (MDV), the causative agent of Marek’s disease (MD), is a highly cell-associated avian alphaherpesvirus that induces immunosuppression, peripheral paralysis and metabolic dysfunction, including increased cholesterol biosynthesis, and transformation of chicken CD4^+^ T cells [1,2,3,4,5,6,7]. MDV causes an economically important disease in chickens, and there is no evidence of MDV infections in humans [8]. Vaccination forms the basis of the current MD control program. MDV replicates and sheds from feather follicle epithelial cells and intermediate epidermal layers of the foot skin in cell-free form into the environment [1,9]. Vaccination induces anti-MDV T cell responses [10,11], which reduce host symptoms and prolong survival, but it fails to inhibit virus replication and shedding [1]. Failure to induce sterile immunity has led to the emergence of more virulent MDV [12]. Thus, there is a need for the development of novel MD control strategies that could be used in combination with MDV vaccines to inhibit virus replication and shedding in infected chickens.

Type I IFNs are secretory cytokines that play a crucial role in combating viral infection and modulating the antiviral immune response [13]. IFN signalling stimulates the expression of a wide array of IFN-stimulated genes (ISGs), including the CH25H gene, which encodes cholesterol 25-hydroxylase, catalysing 25-hydroxycholesterol (25-HC) from cholesterol [14,15]. Alternatively, sterol 27-hydroxylase, encoded by the CYP27A1 gene, catalyses 27-hydroxycholesterol (27-HC). Thus, the oxysterols 25-HC and 27-HC are metabolites of cholesterol containing an additional hydroxyl, epoxide, or ketone group compared to the parental compound and have diverse biological properties, including antiviral properties [16]. CH25H is an ISG in mice, while there is no report suggesting that CYP27A1 is induced by IFNs. In fact, there are some reports suggesting that IFN-ɣ [17] or IFN-α [18] reduces the expression of CYP27A1 in human macrophages. Chicken IFN-α also induce ISGs [19], including CH25H, which can inhibit the replication of avian leukosis virus subgroup J (ALV-J) in vitro [20]. However, the role of other oxysterols in inhibiting virus replication in the avian system is yet unknown.

In this study, we analysed the ability of MDV to stimulate the induction of chicken type I IFNs, CH25H and CYP27A1 in CEF and demonstrated that virulent MDV weakly and transiently induces type I IFNs, CH25H and CYP27A1. The results also confirm that 25-HC and 27-HC can inhibit the replication and spread of MDV in vitro.

## 2. Materials and Methods

### 2.1. CEF Culture and Virus Preparations

CEFs were generated from mixed-sex SPF Valo eggs (Valo Biomedia GmbH) incubated in a Brinsea Ova-Easy 190 incubator at 37 °C for 10 days in ovo. CEFs were seeded at a rate of 1.5 × 10^5^ cells/mL in 24-well plates with growth medium (E199 supplemented with 10% tryptose phosphate broth [TPB], 5% foetal calf serum [FCS], 2.8% deionised water, 0.01% amphotericin B, 10 U/mL penicillin and 10 μg/mL streptomycin) and incubated overnight (38.5 °C at 5% CO_2_). The next day, an 80% confluent monolayer was observed, and growth medium was removed and replaced with maintenance medium (E199 supplemented with 10% TPB, 2.5% FCS, 3.5% cell culture grade sterile water, 0.01% amphotericin B, 10 U/mL penicillin and 10 μg/mL streptomycin) or deprivation medium (E199 supplemented with 10% FCS ± glucose or glutamine). At 14 days post-infection (dpi), spleen cells obtained from chickens infected with MDV were collected and placed on a layer of CEFs for a duration of 7 days. Following the observation of cytopathic effects, the cells associated with MDV-infected CEFs were passaged twice onto fresh CEFs to create virus stocks. The resulting cell-associated virus stock was preserved in liquid nitrogen, utilising medium 199 with Earle’s salts (E199) supplemented with 10% heat-inactivated foetal calf serum (HI-FCS) and 10% dimethyl sulphoxide (DMSO).

### 2.2. Cells and MDV Infection

CEF cells were either mock-infected or infected with a very virulent RB1B strain (100 pfu per 1.5 × 10^5^ cells or a multiplicity of infection [MOI] of 0.0006) in triplicates, harvested at 72 hpi and processed for plaque size or virus titre. 25-HC and 27-HC (Sigma–Aldrich, Dorset, UK) were all reconstituted in ethanol. 25-HC (1 µM) or 27-HC (1 µM) were added to CEF cell cultures infected with RB1B (100 pfu) at the time of infection or 24 hpi, and the cells were fixed for plaque size or harvested with trypsin for plaque titre. Non-toxic concentrations of 25-HC and 27-HC were determined in non-infected CEF cells by staining cells with 7AAD after 72 h of culture and estimating the percentage of live cells using flow cytometry. It is worth noting that none of the treatments affected cell growth, and the cell numbers remained comparable to those of the control condition.

#### 2.2.1. MDV Titre

To assess the infection caused by MDV, the infected cells were quantified by titration onto fresh CEF cells. At 72 h post-infection (hpi), the cells were fixed using a mixture of acetone and methanol in a 1:1 ratio. Subsequently, the fixed cells were incubated with an anti-gB monoclonal antibody, specifically the HB-3 clone. Following a series of washes to remove any unbound antibodies, the cells were exposed to horseradish peroxidase-conjugated rabbit anti-mouse Ig. The detection of viral plaques was achieved through the utilisation of a 3-amino-9-ethylcarbazole (AEC) substrate. Finally, the viral plaques were counted under light microscopy, enabling quantitative analysis of the infection.

#### 2.2.2. Measurement of Virus Plaque Sizes 

In each well, there were an estimated 70 to 100 plaques observed, some of which exhibited fusion with adjacent plaques or were positioned along the well’s periphery. This characteristic made it challenging to measure their individual sizes accurately. To address this issue, we employed a standardised approach in previous studies. We measured the sizes of all plaques that could be reliably measured and compared those results with measurements from 20 randomly selected plaques per well. Our findings demonstrated that the size of 20 plaques from each well provided a representative and reliable average size of measurable plaques. To facilitate this analysis, digital images of 20 individual plaques per well were captured using an inverted light microscope with ×4 magnification. These images were processed using Adobe Photoshop software, and the area tool in ImageJ software was utilised to measure the viral plaques. The resulting data are reported as the average plaque size in square millimeters for each experimental condition.

#### 2.2.3. Quantification of Gene Expression by RTqPCR

CEFs were treated with recombinant chicken IFN-α (Bio-Rad Laboratories Ltd., Watford, UK) or diluent, and the expression of genes was analysed using RTqPCR. To obtain total RNA from the CEFs, TRIzol (Thermo Fisher Scientific, Paisley, UK) was employed for purification, following the recommended protocol of the manufacturer. Subsequently, cDNA synthesis was carried out using the Superscript III first-strand synthesis kit (Thermo Fisher Scientific, Paisley, UK) along with oligo-dT primers. For quantitative real-time PCR analysis, the LightCycler 480 II assay (Roche Diagnostics GmbH, Mannheim, Germany) utilising SYBR green was employed. The PCR procedure consisted of an initial preincubation at 95 °C for 5 min, followed by 40 cycles of denaturation at 95 °C for 20 s and annealing at 55 °C to 64 °C (according to the primer-specific annealing temperature [TA]) for 15 s, with elongation occurring at 72 °C for 10 s. Melt curve analysis was performed by heating to 95 °C for 10 s, cooling to 65 °C for 1 min and then reheating to 97 °C. The relative expression levels were determined by comparing the target gene’s quantification to that of the housekeeping gene, RPLP0 [21]. The LightCycler 480 software (Roche Diagnostics GmbH, Mannheim, Germany) was used to calculate relative expression levels of all genes using the primers outlined in Table 1. The data represent the mean of six biological replicates.

#### 2.2.4. CEF Cell IFN-α-Luc Reporter Assay

Poly (I:C) sodium (Sigma–Aldrich/Merck, Dorset, UK) was dissolved in nuclease-free water and stored at −80 °C. CEF cells were infected with RB1B, as described above. The CEF cells were co-transfected using Lipofectamine 2000 (Thermo Fisher Scientific, Paisley, UK) according to the manufacturer’s instructions, with the firefly reporter plasmid pGL-chIFN-α-Luc and the pRL-TK plasmids expressing Renilla luciferase as the internal reference. These cells were either treated with poly(I:C) sodium (Sigma–Aldrich/Merck, Dorset, UK) or infected with RB1B as described above. The cells were harvested at 24, 48 and 72 h post-stimulation (hps), after which luciferase activity was quantified. Briefly, the cells were lysed by adding 1X luciferase cell culture lysis reagent (Promega, Southampton, UK) and mixed with freshly prepared luciferase assay reagent. The luminescence (RLU) was measured on GloMax-Multi plus Microplate reader instrument (Promega, Southampton, UK). 

#### 2.2.5. Statistical Analysis

All data are presented as mean ± standard deviation (SD) from at least three independent experiments. Quantification was performed using Graph Pad Prism 7 for Windows. The differences between groups in each experiment were analysed using the Kruskal–Wallis test (one-way analysis of variance [ANOVA], nonparametric) with post hoc Dunn’s multiple comparison analysis. Results were considered statistically significant at *p* < 0.05 (*).

## 3. Results

### 3.1. MDV Infection Transiently induces IFN-α 

**Figure 1:**
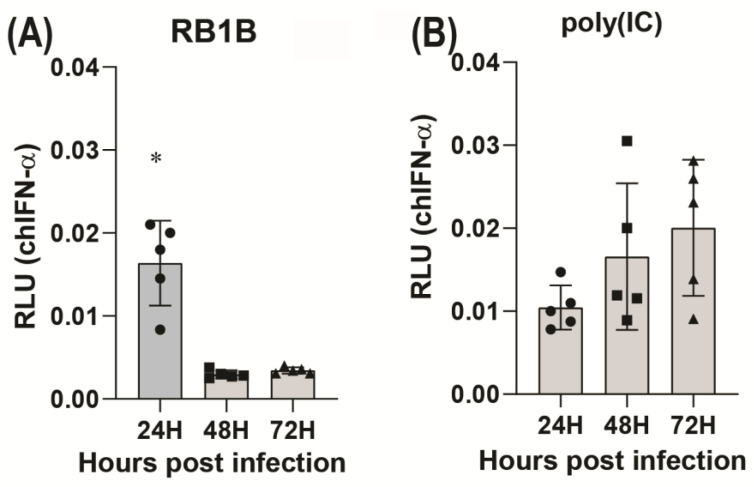
MDV induces a transient IFN-α response. Induction of chicken IFN-α (**A**,**B**) by MDV infection (**A**) or stimulation with poly (I:C) (**B**). CEF cells were transfected with the luciferase gene cloned downstream of the chicken IFN-α promoter. The cells were then either infected with RB1B (100 pfu) or stimulated with poly (I:C) (2.5 μg/mL) for 24, 48 and 72 h. Luciferase assays were performed after 24, 48 and 72 hpi. Relative light units (RLU) at different time points are shown. The data represent three independent experiments, each performed with three biological replicates per treatment. Each dot represents the data from CEFs obtained from embryonated eggs, and the graph shows the mean ± standard deviation of five independent replicates from CEFs obtained from embryonated eggs. Asterisks denote significant differences (*p* < 0.001) by the ANOVA test.

CEF cells were transfected with luciferase gene cloned downstream of chIFN-α promoters. The transfected cells were either infected with MDV (RB1B 100 pfu) (Figure 1A), mock infected or stimulated with poly (I:C) (2.5 μg/mL) (Figure 1B). After 24, 48, and 72 hpi, the cells were analysed for promoter activity by reporter gene assay. The results demonstrated that MDV infection induced chicken IFN-α (Figure 1A) at 24 hpi but reduced at 48 and 72 hpi. In contrast, poly (I:C) treatment increased IFN-α (Figure 1B) responses at all the time points examined in this experiment. The results suggest that MDV infection induces a transient induction of chicken IFN-α in the infected CEF cells.

### 3.2. IFN-α Upregulates CH25H and CYP27A1

**Figure 2:**
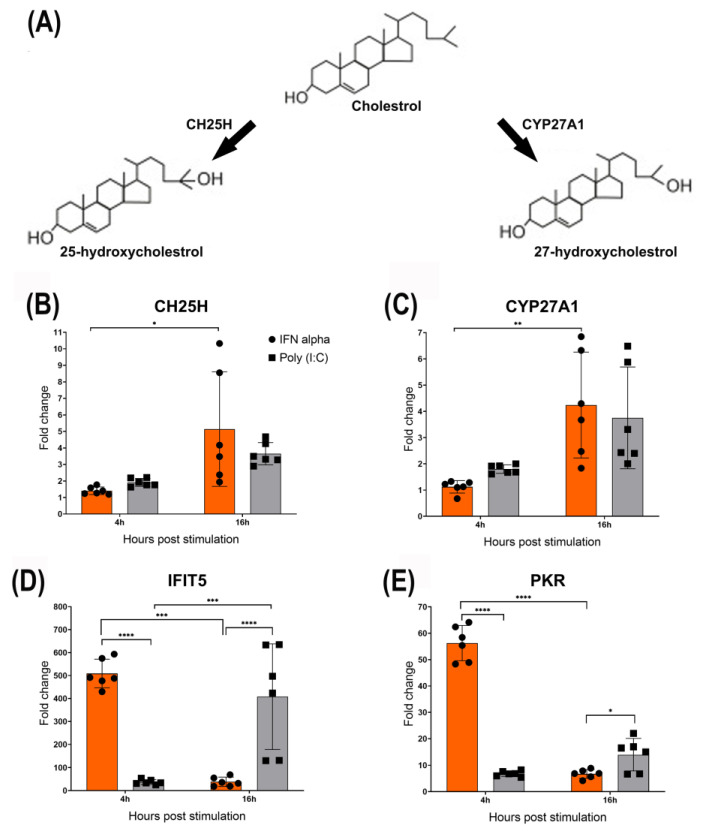
IFN-α upregulates CH25H and CYP27A1. (**A**) A schematic diagram demonstrating that cholesterol can be metabolised into 25-hydroxycholesterol (25-HC) or 27-hydroxycholesterol (27-HC) by the action of the enzyme cholesterol 25-hydroxylase (CH25H) and the cytochrome P450 enzyme oxidase sterol 27-hydroxylase A1 (CYP27A1), respectively. (**B**,**C**) Chicken IFN-α upregulated the expression of CH25H and CYP27A1 in CEFs at 4 hps and significantly increased at 16 hps. Moreover, poly (I:C) was used as a positive control and upregulated both CH25H and CYP27A1 as well, with slight increase over time from 4 hps to 16 hps. (**D**,**E**) IFIT5 and PKR were significantly upregulated at 4 hps compared to 16 hps in IFN-α treated CEFs, and significantly reduced over time from 4 hps to 16 hps. However, with poly (I:C) treatment, IFIT5 significantly increased and PKR showed slight increase over time from 4 hps to 16 hps. In this study, poly (I:C) treatment, with typical ISGs such as IFIT5 and PKR, was used as a control to check the expression of CH25H and CYP27A1 genes. Asterisks denote significant differences (* *p* < 0.05, ** *p* < 0.01, *** *p* < 0.001, **** *p* < 0.0001) by the ANOVA test.

CH25H and CYP27A1 catalyse the production of 25-HC and 27-HC from cholesterol, respectively (Figure 2A). CH25H is an ISG, but there is no report suggesting that IFNs also induce CYP27A1. Here, the results showed that chicken IFN-α upregulated the expression of CH25H and CYP27A1 in CEFs at 4 hps and significantly increased at 16 hps (Figure 2B,C). Moreover, poly (I:C) was used as a positive control and also upregulated both CH25H and CYP27A1, with a slight increase over time from 4 hps to 16 hps (Figure 2B,C). In addition, typical ISGs, such as IFIT5 and PKR, were significantly upregulated at 4 hps compared to 16 hps in IFN-α treated CEFs, and their expression significantly reduced over time from 4 hps to 16 hps (Figure 2D,E). In contrast, with poly (I:C) treatment, IFIT5 increased significantly, and PKR showed a slight increase over time from 4 hps to 16 hps (Figure 2D,E).

### 3.3. MDV Infection Induces Moderate and Transient Upregulation of CH25H and CYP27A1

**Figure 3:**
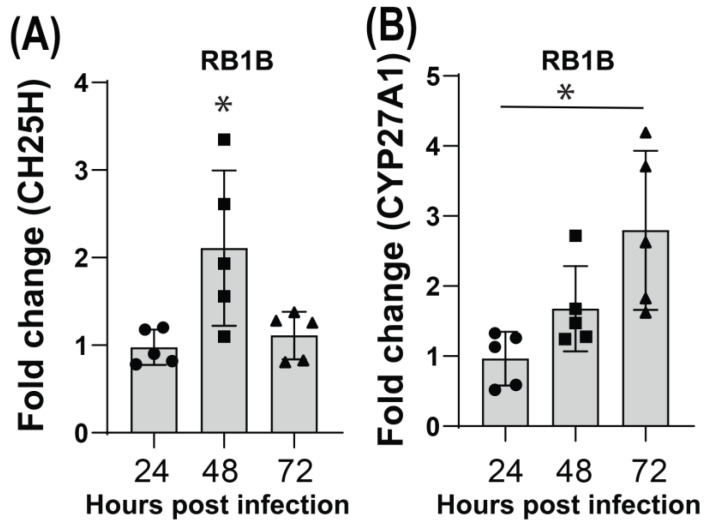
Moderate and transient upregulation of CH25H and CYP27A1 in MDV-infected CEFs. The CEF cells were infected with RB1B (100 pfu) or mock infected, and the expressions of CH25H and CYP27A1 genes were analysed using RT-PCR. (**A**) Transient upregulation of CH25H mRNA level in CEF cells at 48 h post RB1B infection. (**B**) Moderate upregulation of CYP27A1 gene at 72 hpi in CEF cell infected with MDV. The data represent three independent experiments, each performed with three biological replicates per treatment. Each dot represents the data from CEFs obtained from embryonated eggs and the graph shows the mean ± standard deviation of five independent replicates from CEFs obtained from embryonated eggs. Asterisks denote significant differences (*p* < 0.005) by the ANOVA test.

In our experimental setup, we observed that the wild-type RB1B strain of MDV successfully replicates and spreads in vitro, resulting in the formation of visible plaques at 72 h post infection (hpi). Therefore, we conducted an analysis of the expression of CH25H and CYP27A1 at various time points following MDV infection. Interestingly, MDV infection moderately upregulated CH25H at 48 hpi, and the expression of this ISG was reduced to the base level at 72 hpi (Figure 3A). Furthermore, MDV infection only moderately upregulated the expression of CYP27A1 at 72 hpi in the infected CEFs (Figure 3B).

### 3.4. 25-HC and 27-HC Significantly Reduce MDV Replication and Spread In Vitro

**Figure 4:**
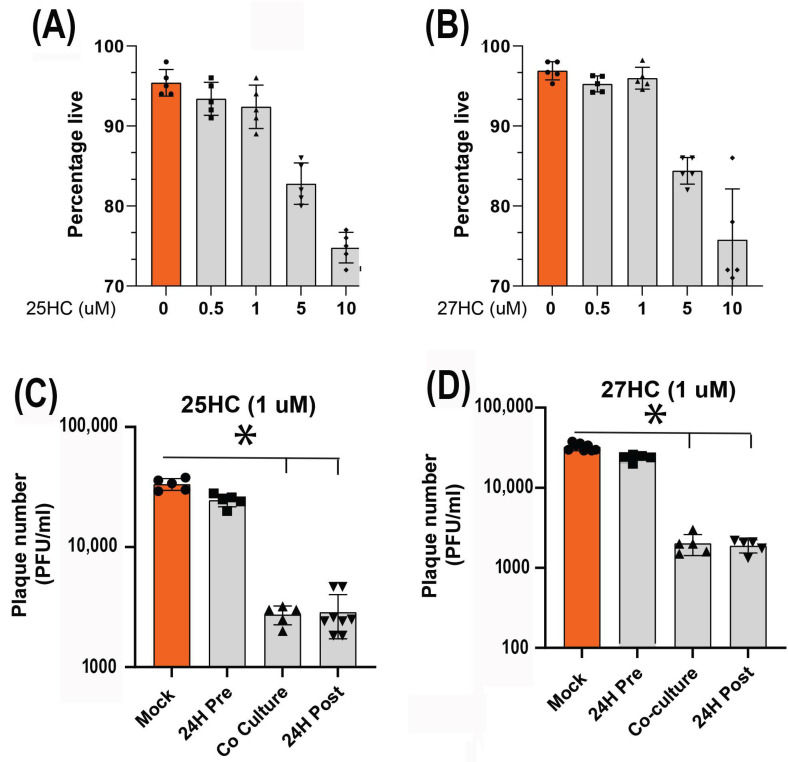
25-HC and 27-HC significantly reduce MDV replication in vitro. (**A**,**B**) Titration of 25-HC and 27-HC for non-toxic concentration in CEF cells calculated by 7-AAD staining and estimated as percentage live population using flow cytometry. The results demonstrated that 1 μM of either 25-HC or 27-HC does not reduce cell viability. (**C**,**D**) 1 μM of either 25-HC or 27-HC was used to assess their effect on MDV titer 72 hpi. CEFs were treated with 25-HC or 27-HC 24 h before infection, at the time of infection or 24 h after infection. Data are a representation of three independent experiments, each performed with three biological replicates per treatment. Each dot represents the data from CEF cells obtained from an embryonated egg and the graph represents the mean ± standard deviation of five independent replicates from individual eggs. Orange color was used for better comparative visualisation between the mock treated and treated groups. Asterisks denote significant differences (*p* < 0.005) by the ANOVA test.

25-HC and 27-HC have been shown to inhibit the replication of both enveloped and non-enveloped human viruses [22], but their role in inhibiting avian viruses is less known. The treatment of CEF cells with different concentrations of 25-HC and 27-HC for 72 h showed that 1 μM of either 25-HC or 27-HC is the highest concentration that is not toxic to the cells (Figure 4A,B). The results of the plaque assay for MDV showed that the treatment of CEF cells at the time of MDV infection or 24 hpi with 1 μM of either 25-HC or 27-HC significantly inhibited MDV titre at 72 hpi. However, the treatment of CEF cells 24 h before MDV infection did not alter MDV titre (Figure 4C,D).

**Figure 5:**
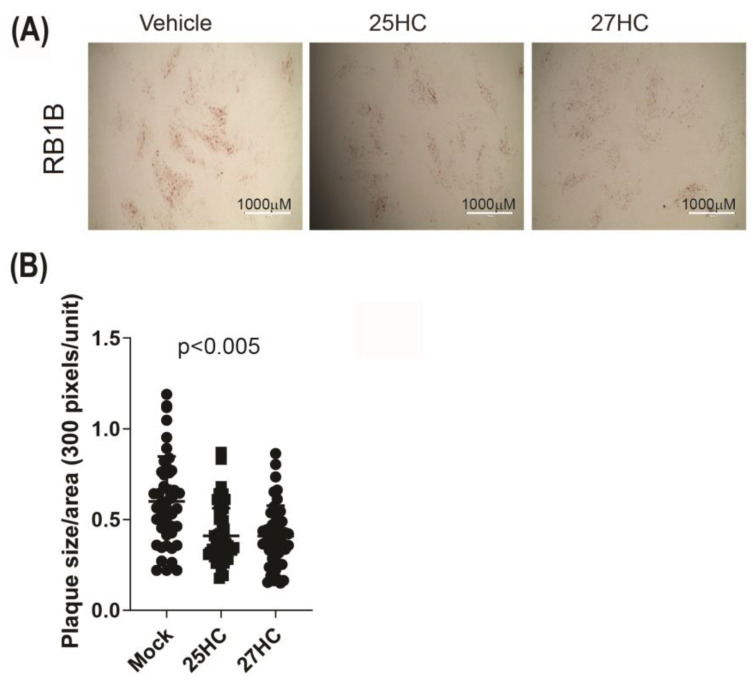
25-HC and 27-HC significantly reduce MDV spread in vitro. Analysis of MDV spread based on plaque size areas (square millimeters) in the presence of 25-HC (1 μM), 27-HC (1 μM) or vehicle at the time of RB1B infection (100 pfu). Plaque sizes were analysed at 72 hpi. (**A**) Representative plaque images are shown. Scale bar, 1000 μm. (**B**) All plaque sizes are shown. Each symbol represents the average sizes of 20 plaques. The experiments were performed in triplicate, and data are representative of 3 independent experiments. ANOVA test showed significant differences between the mock vs. 25-HC- and 27-HC-treated groups (*p* < 0.005).

Plaque sizes were also analysed at 72 hpi. The results demonstrate that treatment of CEF cells with 25-HC or 27-HC at the time of MDV infection significantly reduces plaque sizes (Figure 5A,B).

## 4. Discussion

Vaccinal immunity prolongs the survival of infected chickens but fails to control MDV replication and shedding; as a result, more virulent viruses are emerging from the MDV infected chickens. Thus, other more effective vaccines or control measures should be developed to control MDV replication and spread. The administration of IFN-α has shown promise in reducing MDV replication and prolonging survival of MDV-infected chickens, suggesting that type I IFNs play a role in the control of MDV replication [23,24]. However, these findings also demonstrate that the repeated administration of IFN-α only delayed the onset of the disease but failed to prevent it in MDV-infected chickens [23]. The development of potent antiviral compounds with low toxicity, high stability and, most importantly, low cost is necessary for the control of avian viruses such as MDV. 

25-HC and 27-HC have low toxicity and broad-spectrum antiviral properties against mammalian viruses [25]. It has recently been shown that 25-HC can restrict the replication of the avian leukosis virus subgroup J in vitro [20], suggesting that oxysterols may also be used to control avian viruses. 25-HC regulates viral internalisation and disrupts the maturity of viral proteins mainly by restricting lipid rafts and cholesterol synthesis [25,26]. Additionally, both 25-HC and 27-HC inhibit rotavirus infection by sequestering viral particles into late endosomes [22], and downregulating junction adhesion molecule-A (JAM-A) and the cation-independent isoform of mannose-6-phosphage receptor (MPRci) [27]. This indicates that, overall, 25-HC and 27-HC regulate cholesterol homeostasis and proteome modulation [25,27]. Therefore, it would be interesting to study these mechanisms during MDV infection. We recently showed that MDV infection induces the upregulation of cholesterol biosynthesis, which is essential for MDV replication and spread [7]. Thus, we hypothesise that oxysterols may be used to control MDV replication and spread. The results demonstrate that non-toxic concentrations of 25-HC or 27-HC can effectively control MDV replication in vitro. The antiviral properties of oxysterols have also been confirmed in vivo in murine models, pigs, and rhesus monkeys; however, further research is required to demonstrate the antiviral properties of 25-HC and 27-HC in chickens. In vivo, 25-HC has been successfully used to reduce viremia and the viral load of the highly pathogenic porcine reproductive and respiratory syndrome virus, alleviate lung injury and increase survival rates [28]. Experiments are planned in order to examine the ability of the oxysterols to control MDV replication in both naive and MDV-vaccinated chickens. Moreover, in the past, we reported that 1α,25-dihydroxyvitamin D3 (1α, 25 (OH)2D3; Vitamin D) has immunomodulatory properties on chicken T lymphocytes without inducing unresponsiveness and by limiting immune pathology [29]. Additionally, diet vitamin D3 is hydroxylated into 25(OH)D3 via CYP27A1, which is finally converted into 1α, 25 (OH)2D3 via CYP27B1 and, therefore, it would be interesting to explore the similarities in role between 25-HC and 27-HC.

There has been some contrast between in vitro and in vivo results regarding the ability of MDV to induce type I IFNs. In vitro, it has been suggested that the virulent MDV suppresses IFN-β responses [30,31], while others have shown that the IFN-α gene is upregulated in chickens infected with virulent MDV [32,33]. There is no report suggesting that MDV viral proteins specifically block IFN-β, but not IFN-α, expression in vitro, nor any evidence suggesting that MDV does not induce IFN-β in vivo. The results from in vitro blocking assays using anti-IFN-α antibodies have indicated that MDV infection did not induce bioactive IFN-β [23]. However, our results, for the first time, demonstrate that MDV infection induces the transient upregulation of IFN-α in CEF cells. Further experiments are required to establish any differential induction or inhibition of type I IFNs during MDV infection. 

In our system, chicken IFN-α upregulated the expression of CH25H, an ISG in mice, encoding an enzyme involved in the catalysis of 25-HC from cholesterol. A moderate and transient upregulation of CH25H in MDV-infected CEF cells suggests that MDV is not a potent inducer of CH25H. However, the role of this transient induction of CH25H in the replication of MDV is yet unknown. Inflammatory cytokines (e.g., IL-1β, IL-6, and TNF-α) induced following Zika virus infections in human macrophages also upregulate CH25H in type I IFNs in an independent manner [34]. CH25H does not seem to be a classical ISG in human hepatoma cells, as it cannot be induced by type I IFNs, and it represents a direct innate immune response to viral infection [35]. Our results implicate that chicken IFN-α induces CH25H and CYP27A1 upregulation, thus CH25H is a classical ISG in chicken, and CYP27A1 could be as well. Interestingly, the CYP27A1 gene, involved in the catalysis of 27-HC from cholesterol, was also moderately upregulated at the late stage of MDV infection. The upregulation of CYP27A1 could be attributed to the control of excessive cholesterol biosynthesis at the late stage of in vitro infection. 

Moreover, MDV is a cell-associated virus; high MOI are not possible and, usually, the apparent cytopathic effects and plaques are visible after 48 hpi. This means that, at earlier time points, the majority of cells remain uninfected, which clearly obscures any host gene expression due to MDV infection. Indeed, in the past, we and others have observed that 48 and 72 hpi are ideal for understanding host interactions with MDV [5,6,36]. In addition, it has been reported that IFN-α upregulates ISGs at the early time points, and therefore we evaluated them at 6 and 16 hps to check whether IFN-α upregulates CH25H and CYP27A1 [19,37]. In future work, it would be interesting to measure CH25H and CYP27A1 with virus kinetics at later time points and compare them with in vivo chickens.

In the 1970s and 1980s, numerous compounds were investigated for their activity against in vitro MDV infections. These compounds included pyrophosphate analogues [38], Ribavirin [39], Acyclovir [40] and Fluoropyrimidine nucleosides [41], showing varying degree of effectiveness, ranging from moderate to high. However, when tested against in vivo MDV infections, only a few compounds demonstrated effectiveness. These included Ribavirin and Amino-ureidophenylsulphone (AUS) in reducing MDV lesions [39,42]; (ii) dichlorodiphenyldicholoroethane (DDD) in reducing severity of the disease [43]; Acyclovir in diminishing the development of tumours [44]; and cyclophosphamide, which delayed and reduced delays in viremia and in the development of lesions [45,46]. Recently, Chinese herbs were also screened for their potential against MD [47], and baicalin, a flavonoid, was found to inhibit MDV replication and virus infectivity in vitro in CEFs [48]. Additionally, CRISPR/Cas9-based transgenic chickens expressing Cas9 and gRNA against ICP4 were reported as an effective antiviral approach to control MDV infections in chickens [48]. However, to fully understand the potentials of these compounds, further in vitro and in vivo studies are necessary, including evaluating their cytotoxicity and efficacy using the latest available high-throughput assays.

It is yet to be determined whether these genes are also induced during MDV infection and to examine their role in the pathogenesis of the disease in vivo. Nonhuman primates exhibited tolerance to the intravenous administration of 1.5 mg/Kg/day of 25 HC, resulting in a reduction in viral infection [49]. To assess the toxicity and effectiveness of oxysterols as antiviral agents in birds, in vivo experiments should be conducted. It is unclear whether these compounds could be used locally, for example, in the upper respiratory system, to control respiratory infections in birds. However, it is important to consider the potential unwanted side effects associated with these compounds including inflammation, modulation of IgA production and the promotion of intracellular bacterial growth by the induction of pro-survival factors in macrophages [50,51]. Furthermore, it should be emphasised that the practical use of these oxysterols in the poultry industry may present challenges, including potentially high costs associated with their application. These compounds are not being proposed for direct use in poultry, but rather for the selection of birds expressing high levels of genes associated with the synthesis of these oxysterols in response to viral infections. Moreover, this study utilises MDV infection of chicken cells as a model system. This research could serve as an additional in vitro proof-of-concept for potential application in humans facing herpesvirus infections.

## 5. Conclusions

Here, we demonstrated that MDV infection induces a transient and early IFN-α response, and that IFN-α upregulated both CH25H and CYP2A1. MDV infection also induced the transient and weak upregulation of CH25H, an ISG that catabolises 25-HC oxysterol from cholesterol. CYP27A1, not an ISG that catabolises 27-HC from cholesterol, was transiently induced in the late stage of MDV infection. Both 25-HC and 27-HC reduced MDV titre and spread in vitro. These findings identify two oxysterols with the ability to control MDV replication and to spread and encourage the development and selection of chicken lines expressing genes involved in the synthesis of oxysterols with antiviral properties in response to viral infections.

## Figures and Tables

**Table 1:** List of primers used in this study.

Gene	GenBank Accession No.	Primer Sequence	Tm (°C)	Product Size (bp)
CYP27A1	XM_040676620.2	For-ACCGCCTCCAGCTGATGT	61	133
		Rev-ATCGGGTATTTGCCCTCCTG	59	
CH25H	NM_001277354.1	For-GACCTTCCGTGGTCAACTCA	59	70
		Rev-GGAGATCATGATGCGGTGCT	60	
IFIT5	NM_001320422	For-CTCCCAAATCCCTCTCAACA	62	146
		Rev-CCGGTCATCGTCTGCATATT	62	
PKR	AB125660.1	For-CAGGCGTTGGTAAGAGTAAGAA	62	135
		Rev-CATCCGCAGGTAGAGGAGATA	62	
Actin-beta	NM_205518.2	For-CCGTGCTGTGTTCCCATCTA	59	98
		Rev-TCTGGGCTTCATCACCAACG	60	
RPLP0	NM_204987	For-TTGGGCATCACCACAAAGATT	65	82
		Rev-CCCACTTTGTCTCCGGTCTTAA	67	

## Data Availability

The data are contained within the article.

## References

[1] Boodhoo N., Gurung A., Sharif S., Behboudi S. (2016). Marek’s disease in chickens: A review with focus on immunology. Vet. Res..

[2] Kamble N., Gurung A., Kaufer B.B., Pathan A.A., Behboudi S. (2021). Marek’s Disease Virus Modulates T Cell Proliferation via Activation of Cyclooxygenase 2-Dependent Prostaglandin E2. Front. Immunol..

[3] Osterrieder N., Kamil J.P., Schumacher D., Tischer B.K., Trapp S. (2006). Marek’s disease virus: From miasma to model. Nat. Rev. Microbiol..

[4] Nair V. (2013). Latency and tumorigenesis in Marek’s disease. Avian Dis..

[5] Boodhoo N., Kamble N., Sharif S., Behboudi S. (2020). Glutaminolysis and Glycolysis Are Essential for Optimal Replication of Marek’s Disease Virus. J. Virol..

[6] Boodhoo N., Kamble N., Kaufer B.B., Behboudi S. (2019). Replication of Marek’s Disease Virus Is Dependent on Synthesis of De Novo Fatty Acid and Prostaglandin E(2). J. Virol..

[7] Boodhoo N., Kamble N., Behboudi S. (2020). De Novo Cholesterol Biosynthesis and Its Trafficking in LAMP-1-Positive Vesicles Are Involved in Replication and Spread of Marek’s Disease Virus. J. Virol..

[8] Hennig H., Osterrieder N., Muller-Steinhardt M., Teichert H.M., Kirchner H., Wandinger K.P. (2003). Detection of Marek’s disease virus DNA in chicken but not in human plasma. J. Clin. Microbiol..

[9] Lantier I., Mallet C., Souci L., Larcher T., Conradie A.M., Courvoisier K., Trapp S., Pasdeloup D., Kaufer B.B., Denesvre C. (2022). In vivo imaging reveals novel replication sites of a highly oncogenic avian herpesvirus in chickens. PLoS Pathog..

[10] Boodhoo N., Behboudi S. (2022). Marek’s disease virus-specific T cells proliferate, express antiviral cytokines but have impaired degranulation response. Front. Immunol..

[11] Boodhoo N., Behboudi S. (2021). Differential Virus-Specific IFN-Gamma Producing T Cell Responses to Marek’s Disease Virus in Chickens With B19 and B21 MHC Haplotypes. Front. Immunol..

[12] Read A.F., Baigent S.J., Powers C., Kgosana L.B., Blackwell L., Smith L.P., Kennedy D.A., Walkden-Brown S.W., Nair V.K. (2015). Imperfect Vaccination Can Enhance the Transmission of Highly Virulent Pathogens. PLoS Biol..

[13] Teijaro J.R. (2016). Type I interferons in viral control and immune regulation. Curr. Opin. Virol..

[14] Liu S.Y., Sanchez D.J., Aliyari R., Lu S., Cheng G. (2012). Systematic identification of type I and type II interferon-induced antiviral factors. Proc. Natl. Acad. Sci. USA.

[15] Holmes R.S., Vandeberg J.L., Cox L.A. (2011). Genomics and proteomics of vertebrate cholesterol ester lipase (LIPA) and cholesterol 25-hydroxylase (CH25H). 3 Biotech..

[16] Cyster J.G., Dang E.V., Reboldi A., Yi T. (2014). 25-Hydroxycholesterols in innate and adaptive immunity. Nat. Rev. Immunol..

[17] Reiss A.B., Awadallah N.W., Malhotra S., Montesinos M.C., Chan E.S., Javitt N.B., Cronstein B.N. (2001). Immune complexes and IFN-gamma decrease cholesterol 27-hydroxylase in human arterial endothelium and macrophages. J. Lipid Res..

[18] Ahmed D., Jaworski A., Roy D., Willmore W., Golshani A., Cassol E. (2018). Transcriptional Profiling Suggests Extensive Metabolic Rewiring of Human and Mouse Macrophages during Early Interferon Alpha Responses. Mediat. Inflamm..

[19] Giotis E.S., Robey R.C., Skinner N.G., Tomlinson C.D., Goodbourn S., Skinner M.A. (2016). Chicken interferome: Avian interferon-stimulated genes identified by microarray and RNA-seq of primary chick embryo fibroblasts treated with a chicken type I interferon (IFN-alpha). Vet. Res..

[20] Xie T., Feng M., Dai M., Mo G., Ruan Z., Wang G., Shi M., Zhang X. (2019). Cholesterol-25-hydroxylase Is a Chicken ISG That Restricts ALV-J Infection by Producing 25-hydroxycholesterol. Viruses.

[21] Staines K., Batra A., Mwangi W., Maier H.J., Van Borm S., Young J.R., Fife M., Butter C. (2016). A Versatile Panel of Reference Gene Assays for the Measurement of Chicken mRNA by Quantitative PCR. PLoS ONE.

[22] Civra A., Francese R., Gamba P., Testa G., Cagno V., Poli G., Lembo D. (2018). 25-Hydroxycholesterol and 27-hydroxycholesterol inhibit human rotavirus infection by sequestering viral particles into late endosomes. Redox Biol..

[23] Bertzbach L.D., Harlin O., Härtle S., Fehler F., Vychodil T., Kaufer B.B., Kaspers B. (2019). IFNα and IFNγ Impede Marek’s Disease Progression. Viruses.

[24] Jarosinski K.W., Jia W., Sekellick M.J., Marcus P.I., Schat K.A. (2001). Cellular responses in chickens treated with IFN-alpha orally or inoculated with recombinant Marek’s disease virus expressing IFN-alpha. J. Interferon Cytokine Res..

[25] Mao S., Ren J., Xu Y., Lin J., Pan C., Meng Y., Xu N. (2022). Studies in the antiviral molecular mechanisms of 25-hydroxycholesterol: Disturbing cholesterol homeostasis and post-translational modification of proteins. Eur. J. Pharmacol..

[26] Campbell S.M., Crowe S.M., Mak J. (2001). Lipid rafts and HIV-1: From viral entry to assembly of progeny virions. J. Clin. Virol..

[27] Civra A., Colzani M., Cagno V., Francese R., Leoni V., Aldini G., Lembo D., Poli G. (2020). Modulation of cell proteome by 25-hydroxycholesterol and 27-hydroxycholesterol: A link between cholesterol metabolism and antiviral defense. Free Radic. Biol. Med..

[28] Song Z., Bai J., Nauwynck H., Lin L., Liu X., Yu J., Jiang P. (2019). 25-Hydroxycholesterol provides antiviral protection against highly pathogenic porcine reproductive and respiratory syndrome virus in swine. Vet. Microbiol..

[29] Boodhoo N., Sharif S., Behboudi S. (2016). 1alpha,25(OH)2 Vitamin D3 Modulates Avian T Lymphocyte Functions without Inducing CTL Unresponsiveness. PLoS ONE.

[30] Gao L., Li K., Zhang Y., Liu Y., Liu C., Zhang Y., Gao Y., Qi X., Cui H., Wang Y. (2019). Inhibition of DNA-Sensing Pathway by Marek’s Disease Virus VP23 Protein through Suppression of Interferon Regulatory Factor 7 Activation. J. Virol..

[31] Liu Y., Gao L., Xu Z., Luo D., Zhang Y., Gao Y., Liu C., Zhang Y., Qi X., Cui H. (2019). Marek’s Disease Virus RLORF4 Inhibits Type I Interferon Production by Antagonizing NF-κB Activation. J. Virol..

[32] Abdul-Careem M.F., Hunter B.D., Lee L.F., Fairbrother J.H., Haghighi H.R., Read L., Parvizi P., Heidari M., Sharif S. (2008). Host responses in the bursa of Fabricius of chickens infected with virulent Marek’s disease virus. Virology.

[33] Jarosinski K.W., Njaa B.L., O’Connell P.H., Schat K.A. (2005). Pro-inflammatory responses in chicken spleen and brain tissues after infection with very virulent plus Marek’s disease virus. Viral Immunol..

[34] Magoro T., Dandekar A., Jennelle L.T., Bajaj R., Lipkowitz G., Angelucci A.R., Bessong P.O., Hahn Y.S. (2019). IL-1β/TNF-α/IL-6 inflammatory cytokines promote STAT1-dependent induction of CH25H in Zika virus-infected human macrophages. J. Biol. Chem..

[35] Xiang Y., Tang J.J., Tao W., Cao X., Song B.L., Zhong J. (2015). Identification of Cholesterol 25-Hydroxylase as a Novel Host Restriction Factor and a Part of the Primary Innate Immune Responses against Hepatitis C Virus Infection. J. Virol..

[36] Morgan R.W., Sofer L., Anderson A.S., Bernberg E.L., Cui J., Burnside J. (2001). Induction of host gene expression following infection of chicken embryo fibroblasts with oncogenic Marek’s disease virus. J. Virol..

[37] Qu H., Yang L., Meng S., Xu L., Bi Y., Jia X., Li J., Sun L., Liu W. (2013). The differential antiviral activities of chicken interferon alpha (ChIFN-alpha) and ChIFN-beta are related to distinct interferon-stimulated gene expression. PLoS ONE.

[38] Reno J.M., Lee L.F., Boezi J.A. (1978). Inhibition of herpesvirus replication and herpesvirus-induced deoxyribonucleic acid polymerase by phosphonoformate. Antimicrob. Agents Chemother..

[39] Eidson C.S., Than V.T., Kleven S.H. (1974). The in vitro and in vivo effect of chemotherapeutic agents on the Marek’s disease herpesvirus of chickens. Poult. Sci..

[40] Collins P. (1983). The spectrum of antiviral activities of acyclovir in vitro and in vivo. J. Antimicrob. Chemother..

[41] Schat K.A., Schinazi R.F., Calnek B.W. (1984). Cell-specific antiviral activity of 1-(2-fluoro-2-deoxy-beta-D-arabinofuranosyl)-5-iodocytosine (FIAC) against Marek’s disease herpesvirus and turkey herpesvirus. Antivir. Res..

[42] Chang T.S. (1984). AUS in the prevention of Marek’s disease. Avian Dis..

[43] Colmano G., Gross W.B. (1971). Effect of metyrapone and DDD on infectious diseases. Poult. Sci..

[44] Samorek-Salamonowicz E., Cakala A., Wijaszka T. (1987). Effect of acyclovir on the replication of turkey herpesvirus and Marek’s disease virus. Res. Vet. Sci..

[45] Lu Y.S., Kermani-Arab V., Moll T. (1976). Cyclophosphamide-induced amelioration of Marek’s disease in Marek’s disease-susceptible chickens. Am. J. Vet. Res..

[46] Kermani-Arab V., Moll T., Cho B.R., Davis W.C., Lu Y.S. (1975). Effect of cyclophosphamide on the response of chickens to a virulent strain of Marek’s disease virus. Infect. Immun..

[47] Sun Y., Niu L., Song M., Zhao X., Sun N., He J., Wu C., Jiang J., Bai Y., Guo J. (2014). Screening compounds of Chinese medicinal herbs anti-Marek’s disease virus. Pharm. Biol..

[48] Yang F., Feng C., Yao Y., Qin A., Shao H., Qian K. (2020). Antiviral effect of baicalin on Marek’s disease virus in CEF cells. BMC Vet. Res..

[49] Li C., Deng Y.Q., Wang S., Ma F., Aliyari R., Huang X.Y., Zhang N.N., Watanabe M., Dong H.L., Liu P. (2017). 25-Hydroxycholesterol Protects Host against Zika Virus Infection and Its Associated Microcephaly in a Mouse Model. Immunity.

[50] Trindade B.C., Ceglia S., Berthelette A., Raso F., Howley K., Muppidi J.R., Reboldi A. (2021). The cholesterol metabolite 25-hydroxycholesterol restrains the transcriptional regulator SREBP2 and limits intestinal IgA plasma cell differentiation. Immunity.

[51] de Freitas F.A., Levy D., Reichert C.O., Cunha-Neto E., Kalil J., Bydlowski S.P. (2022). Effects of Oxysterols on Immune Cells and Related Diseases. Cells.

